# Mr. Jenkinson on the Cure of Rheumatism

**Published:** 1804-06-01

**Authors:** John Jenkinson

**Affiliations:** Manchester


### *To the Editors of the Medical and Phyſical Journal.*

Gentlemen,

I HAVE for some time intended to give you an account of the efficacy of arsenic, internally administered in chronic rheumatism; a disease which it has never before, to the best of my knowledge, been employed to relieve; and I have only been waiting for more experience, to justify my recommendation of it. Having in the course of last summer, summer, unsuccessfully exhausted the common list of anti-rheumatic remedies (warm bath, various liniments, antimony, Dover's powder, bark, mercury, opium, guaiacum and ammonia, in several forms and combinations; mustard seed, horse-raddish, &c. &c.) in the case of a friend of mine, who was every day becoming a greater martyr to that tormenting complaint, some analogies and reasonings, which will probably appear very loose to many of your readers, and particularly to those who do not know how casually medical discoveries are often brought to light, led me to the use of this medicine; I will slightly advert to them before I proceed.

1. Most of the common anti-rheumatic remedies; for instance, warm bathing, antimony, guaiacum, mercury, bardana, are employed in cutaneous diseases: arsenic too, is sometimes so employed.

2. Arsenic cures certain intermitting paroxysms and pains, as ague, and head-ache; the pains of rheumatism have their periodical aggravations.

3. Arsenic is a powerful tonic, and chronic rheumatism is a disease of debility.

The sufferings of my patient, a young man of twenty-six, were altogether confined to his chest and loins for several months; he was during this time nearly free from pain in the day, but the nightly paroxysms kept increasing in violence. His limbs at length became generally affected, and one of his knuckles much enlarged; he was now never easy upon motion, and at last got almost unable to leave his room. His appetite and strength were much impaired, and having before tried such a variety of remedies, he despaired of his situation, and began to set himself down as a cripple for life. He had, moreover, been troubled for some years with a pretty general eruption of the herpetic kind, which first appeared after a course of mercury, and which at times was very virulent. Under these circumstances it was, that I recommended to him the use of Fowler's mineral solution: he began, as is usual, by taking four drops in water, three times a day, which he gradually increased to three, and finally to four times the number; a quantity which few persons can bear, ten or twelve drops being commonly sufficient. In two or three weeks, without the aid of any other remedy, he was considerably relieved, and by a perseverance of two months in the course, was, and still remains, perfectly cured, both of rheumatism and herpes. He began with the solution in October, and consequently recovered under all the disadvantages disadvantages of wet, cold, and changeable weather. As he was completely tired of nauseous medicines before, the tastelessness of this was a great recommendation with him, as it must be with others, and contributed very much to his steadiness in its use. I have since had the satisfaction of relieving other rheumatic patients by the same means, after the failure of the usual remedies; and Mr. Hardman (the gentleman who lately gave you an account of his success with the cupping glass, in a case of abscess) informs me, that a lady under his care has lately been relieved from the most violent pains of the kind, which had set the best of the old remedies at defiance for thirteen or fourteen months, by the use of the mineral solution alone. The disease began, in this case, to yield to it in three or four days; she still continues the medicine, and has lately walked three or four miles at a time, after having been unable to move about her own house with comfort for several months. In this case too, as in the others, enlargements of the joints have subsided, and I have no doubt from what I have seen, that this remedy, employed with proper discrimination and care, will be found worthy to supersede many others, and to afford more relief than has commonly been afforded in the worst cases of chronic rheumatism. I must be allowed to make two or three remarks before I conclude.

It will be thought by some, that the cutaneous disease led me to the use of the arsenic in the first case. I should not deem such a remark of any great consequence, if it were urged; but I will just assure you that it never occured to me, in the first instance, to direct my attention to the removal of an eruption, which length of time had rendered familiar, and to the disappearance of which every subsequent illness might have been ascribed, as is occasionally done; and that this circumstance added but little to my previous determination to employ that solution on account of the rheumatism, though its double effect has certainly much increased the pleasure of the cure. Lest the union of two complaints in this case may lead to some suspicion of the pain differing in any respect from rheumatic pain in general, it may be worth while to observe, that in the other cases there was nothing of the kind.

Cases of chronic rheumatism, in which the usual remedies have failed, and in which the arsenic may be improper, will no doubt occur; but with your readers, I conceive particular references are quite unnecessary: most gentlemen gentlemen of the profession are fully competent to form their own judgments. For the same reason, it may be superfluous to hint, that a few drops of laudanum will be occasionally required, to make the solution sit easy with delicate persons, and that a gentle laxative, in case of costiveness during its use, should not be forgotten. Henceforth, perhaps, it may be right to give the solution a trial, for reasons obvious to your readers, in some tedious arthritic cases; and likewise in the wandering pains and anomalous ailments of those syphilitic patients, who have sustained permanent injury both from the disease and its cure. It would be easy to extend the prospect still farther, but let us first cultivate the ground we have gained.

A professional gentleman, to whom I mentioned part of the facts here detailed, gave it as his opinion, that the arsenic owed its success in rheumatism *entirely* to its tonic powers. Undoubtedly it would not have so succeeded, if its powers had been of an opposite kind; but it is not the property of tonics in general, *directly* to relieve rheumatic pains, and reduce rheumatic enlargements; nor can we attribute to its tonic powers its alleged anodyne properties in some cancerous cases. Besides, the pain in all the patients alluded to in this letter, has yielded to it before the strength was perceptibly, or could be materially improved, insomuch, that the strength may as well be called the consequence of the ease as the reverse. That arsenic has a local, in addition to a general effect, is evident from the itching and partial discolorations so often attendant upon its use; and it is certainly to its peculiar action upon the superficial circulation, that we must ascribe much of its efficacy in cutaneous complaints, if not its other qualities However, I am by no means inclined to be uneasy about its modus operandi, I am only anxious about the matter of fact; and I hope, on that account, that your Correspondents will turn their attention to the subject without delay: I have much deceived myself, if it be not very well worth their while.

I wish most sincerely I may soon have more success to communicate: in the mean time, I shall be particularly obliged to any gentleman who publishes an account of impartial trials made with this medicine, be the event what it may.

I am, &c.

JOHN JENKINSON.

*Manchester, May* 21, 1804.

